# An Efficient and Chemoselective Procedure for Acylal Synthesis

**DOI:** 10.3390/molecules15096493

**Published:** 2010-09-16

**Authors:** Da-He Fan, Hui Wang, Xing-Xing Mao, Yong-Miao Shen

**Affiliations:** 1 School of Chemical and Biological Engineering, Yancheng Institute of Technology, Yancheng 224003, China; 2 Department of Chemistry, Shaoxing University, Shaoxing 312000, China

**Keywords:** efficient, novel catalyst, heterogeneous, green process

## Abstract

A novel heterogeneous efficient procedure has been developed for the chemoselective synthesis of acylals (1,1-diacetates) under solvent-free conditions. A novel catalyst prepared by the sulfuric acid catalyzed copolymerization of *p*-toluenesulfonic acid and paraformaldehyde displays extremely high activities for the title reactions, affording average yields over 90% within several minutes. A comparative study showed that the novel catalyst has much higher activity than other catalysts used for this purpose. Besides, the novel catalyst displays chemoselectivity for the protection of aldehydes in the presence of ketones. In addition the high acidity (4.0 mmol/g), thermal stability (200 ºC) and easy reusability make the novel catalyst one of the best choices for the process.

## 1. Introduction

**Scheme 1:**
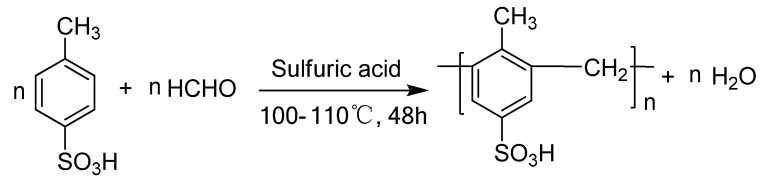
The synthetic route to the novel catalyst.

Acylals (1,1-diacetates) are widely utilized as one of the most useful protecting groups for carbonyl compounds because of their stability under various reaction conditions and their easy conversion back to the parent compounds [1]. Beyond their protecting role, acylals are also important precursors for the synthesis of dienes, chiral allyclic esters and as cross linking reagents for the cellulose in cotton [2,3,4,5], hence methods for their synthesis have received considerable attention. Some of the reported catalysts for the preparation of 1,1-diacetates from aldehydes and acetic anhydride include Sc(OTf)_3_ [6], PCl_3_ [7], TMSCl (trimethylchlorosilane)-NaI [8], iodine [9], sulfuric acid [10], PVA-FeCl_3_ [11], NBS (*N*‑bromosuccinimide) [12], triflic acid [13], Cu(OTf)_3_ [14], CAN (ceric ammonium nitrate) [15], ZrCl_4 _[16] and Bi(OTf)_3 _[17]. Although some of these methods offer convenient protocols with good to high yields, the majority suffer from at least one of the following disadvantages: reactions under oxidizing conditions, use of halogenated solvents, high temperatures, long reaction times, moisture sensitivity of the catalyst, high cost and high toxicity. The well-documented advantages of solid catalysts and solvent-free reactions are safety, economy, high yields, easy workup procedures and short reaction times. Aiming for these advantages, many heterogenous catalysts such as zinc-montmorillonite [18], SnCl_4_/SiO_2_ [19], zirconium sulfate tetrahydrate-silica gel [20], SO_3_H-functionalized ionic liquids [21], silica-bonded *S*-sulfonic acid [22], silica-supported perchloric acid (HClO_4_-SiO_2_) [23], copper *p*-toluenesulfonate/HOAc [24], H_3_PW_12_O_40_ supported MCM-41 [25] and solid superacids [26] have been used for the synthesis of acylals. Here we report the development of a solvent-free protocol for the conversion of aldehydes into acylals using a novel heterogeneous acid catalyst that has been synthesized through the sulfuric acid catalyzed copolymerization of *p*-toluenesulfonic acid (PTSA) and paraformaldehyde (Scheme 1). The results showed that the thus prepared catalyst displays very high activity for the target reactions, affording average yields over 90% within several minutes. A comparative study showed that the novel catalyst has much higher activity than other catalysts ued for this reaction, with the additional advantage of reusability. The novel catalyst also displays chemoselectivity for the protection of aldehydes in the presence of ketones.

## 2. Results and Discussion

### 2.1. Characterization of the novel catalyst

**Figure 1:**
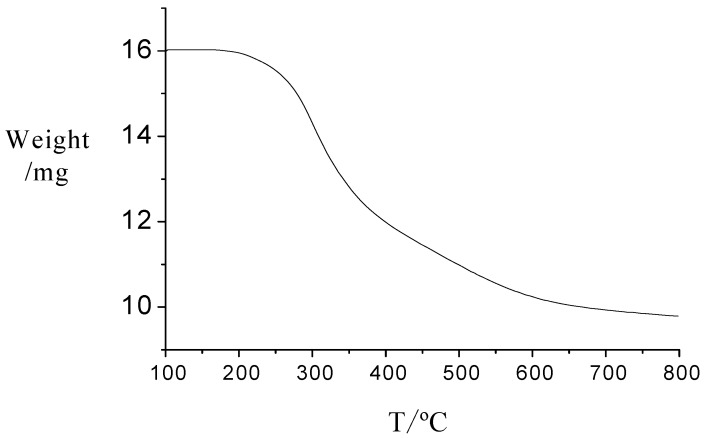
The TG curve of the novel catalyst.

The acidity of the novel catalyst was determined through a neutralization titration which gave the results of 4.0 mmol/g, a little lower than that of pure PTSA (5.8 mmol/g). The catalyst showed much higher acidity than other common heterogeneous acid catalysts such as Nafion and Amberlyst-15 (0.8 mmol/g). It can be seen from Scheme 1 that the acidity of the catalyst originated from the sulfonic group of the PTSA molecule, so the acidic sites in the novel catalyst were strong acid sites, which was confirmed by thermodesorption of chemisorbed ammonia (NH_3_-TPD). The result showed that the catalyst had high acid strength in which ammonia desorbed in the range from 673 to 873 K a little higher than that of the pure PTSA. NH_3_-TPD gave an acidity of 5.0 mmol/g, which indicated that many acid sites were hiding inside the novel catalyst. The BET surface area using nitrogen adsorption isotherms at the temperature of liquid nitrogen gave the result of 23 m^2^/g which also support the results above. It can also be seen from Scheme 1 that almost every benzyl ring was attached with the sulfonic groups. As a result, the catalyst should in theory display an acidity as high as 5.4 mmol/g. Here some sulfonic groups might fall off from the PTSA molecule and not only the linear chains but also branches or cross-linked structures could be formed during the process. That was in accordance with the results of the elemental analysis, which gave the result: C 54.3%; H 5.3%; S 16.2% compared to the theoretical results: C 52.1%; H 4.3%; S 17.4%, indicating that almost all the S element existed in the catalyst in the form of sulfonic acid groups, which also confirmed the strong acid sites equal to the results of NH_3_-TPD. The carbon and hydrogen content was a little more than the theoretical value, which indicated the presence of more CH_2_ groups in the catalyst, possibly in the form of some branches or cross-linked structures. The thermal stability of the catalyst is an important property. The TG curve for the catalyst is shown in Figure 1. The decrease in weight began at about 200 ºC, indicating the decomposition of the catalyst. The covalent chemical bonds connection made the catalyst highly thermally stable. This suggests that the catalyst could be used for high temperature applications. 

**Figure 2:**
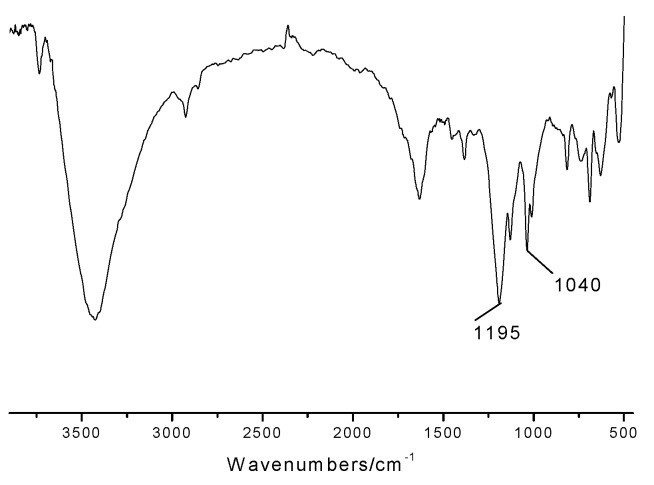
The IR spectrum of the novel catalyst.

The IR spectrum of the catalyst (Figure 2) showed the expected sulfonic group absorbtions at 1,040 and 1,195 cm^-1^ which confirmed the existence of the sulfonic acid groups.

### 2.2. Catalytic procedure for the synthesis of acylals

**Scheme 2:**
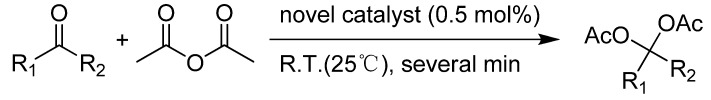
The reaction scheme.

A wide variety of carbonyl compounds were successfully transformed into the corresponding acylals using the novel catalyst under solvent-free conditions (Scheme 2). It can be seen from Table 1 that the catalyst was very efficient in these reactions, giving an average yield above 90% within several minutes. It can also be deduced that the reactivity of aromatic aldehydes towards the formation of acylals depends on the substituents present in the aromatic nucleus. Benzaldehyde or aromatic aldehydes containing electron withdrawing substituents attached to the aromatic ring reacted very fast to furnish acylals due to the better electrophilicity at the carbonyl center compared to the aromatic aldehydes with electron donating substituents, which may reduce the electophilicity at the carbonyl center by virtue of conjugation (entries 1-13). The yield of diethylaminobenzaldehyde is only 45% and almost no side reactions occurred under the mild conditions (entry 13). In addition, the formation of acylals from aliphatic aldehydes was also fast, but much slower than that of the aromatic aldehydes (entries 16-18). We have found that both carbonyl and phenolic –OH groups were acylated. The formation of triacetate was observed due to the acylation of phenolic –OH group (entries 9, 10).

**Table 1:** The results with various carbonyl compounds.

Entry	Substrate	Product	Reaction time/min	Yield/% ^a, b^
1	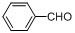	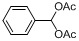	3	98.9
2	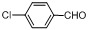	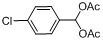	4	97.0
3	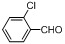	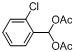	6	94.0
4	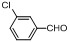	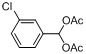	5	95.0
5	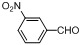	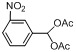	2	99.4
6	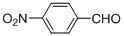	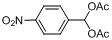	3	99.2
7	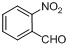	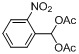	4	99.0
8	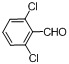	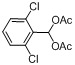	15	98.0
9	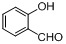	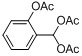	6	94.0
10	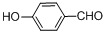	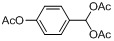	5	95.0
11	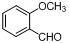	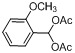	6	94.0
12	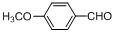	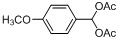	5	95.0
13	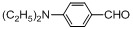	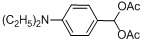	35	45.0
14	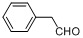	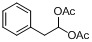	10	90.0
15	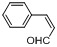	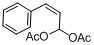	12	91.0
16	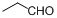	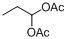	16	92.0
17	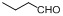	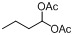	18	90.0
18	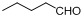	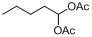	23	90.0

a) The reactions were carried out at R.T. (25 ºC) under solvent free condition: carbonyl compounds 0.02 mol, acetic anhydride 0.04 mol, catalyst 25 mg; b) The yields are isolated yields.

### 2.3. Comparative study on the catalytic activities of the novel catalyst and other catalysts

A comparative study on the catalytic activities of the novel catalyst with some of the reported catalyst was carried out using benzaldehyde as a model substrate (Table 2). From this study it can be concluded that the novel catalyst has much higher activity than others, furthermore it has the added advantage of reusability (entries 1-10). The novel catalyst was also more efficient than the traditional solid acids such as HY zeolites and SO_4_^2-^/TiO_2_ (entries 11 and 12) and the highest yield was achieved with low catalyst usage and short reaction times. This clearly shows that the novel catalyst should be considered as one of the best choices as an economical, convenient, user-friendly catalyst and for scale-up purposes.

**Table 2:** Comparison of different catalysts.

Entry	Catalyst	Catalyst loading/mol%	Reaction time/min	Yield/% ^a, b^
1	H_2_SO_4_	1	5	88.5
2	PTSA	1	5	87.4
3	FeCl_3_	1	5	84.3
4	NH_2_SO_3_H	1	14	88.4
5	Bi(OTf)_3_	1	15	90.3
6	ZrCl_4_	1	8	85.4
7	Iodine	1	25	80.5
8	NBS	1	13	83.2
9	Cu(OTf)_3_	1	10	85.6
10	CAN	1	15	87.8
11	HY	70 mg	30	82.3
12	SO_4_^2-^/TiO_2_	80 mg	15	90.3
13	Novel catalyst	25 mg	3	98.9

a) The reactions were carried out at R.T. (25 ºC) under solvent free condition: benzaldehyde 0.02 mol, acetic anhydride 0.04 mol; b) The yields are isolated yields.

### 2.4. The reuse of the catalyst

**Figure 3:**
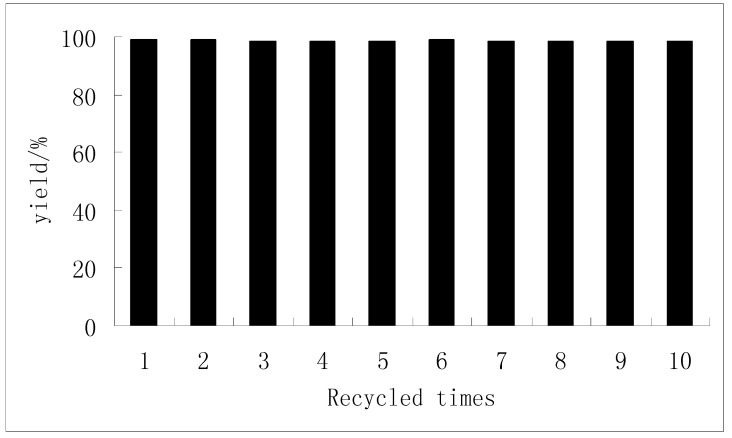
Catalyst reuse.

One interesting property of the novel catalyst is its heterogeneous catalytic process. Thus, recovery of the catalyst is very convenient. After the reactions, the catalyst was recovered by filtration. The activities of the recovered catalyst were carefully investigated through the reaction of benzaldehyde and acetic anhydride (Figure 3). The yields and the sample composition remained unchanged even after the catalyst had been recycled for a tenth time.

### 2.5. The chemoselectivity of the catalyst

**Scheme 3:**
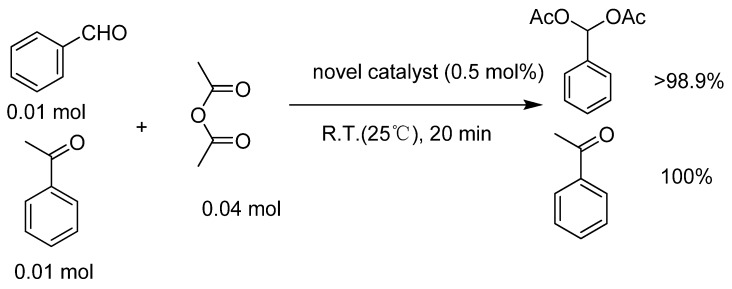
The chemoselectivity of the catalyst.

It is noteworthy that ketones did not produce the corresponding acylals under the same reaction conditions (Scheme 3). This result indicated that the present protocol could be applicable to the chemoselective protection of aldehydes in the presence of ketones.

## 3. Experimental

### 3.1. General

All organic reagents were commercial products with the highest purity available (>98%) and used for the reactions without further purification.

### 3.2. Synthesis of the catalyst

The catalyst was synthesized through the polymerization of *p*-toluenesulfonic acid (PTSA) and paraformaldehyde catalyzed by sulfuric acid (Scheme 1). In the typical procedure a mixture of *p*-toluenesulfonic acid (10 g), paraformaldehyde (2 g) and sulfuric acid (0.2 g) was heated in a three necked round bottomed flask equipped with a magnetic stirrer, a thermometer and a funnel. The reaction mixture was kept in the range of 100-110 ºC for 48 h to form a black solid. Sulfuric acid is absolutely necessary here and the polymerization does not take place efficiently without it. The solid was filtered and washed with hot water (>80 ºC) until no SO_4_^2-^ was detectable in the filtrate. The catalyst was obtained after drying at 120 ºC in an oven overnight. The titration was carried out as follows: 40 mg novel catalyst and 2 N 4 mL aqueous NaCl were stirred at room temperature for 24 h. The solids were filtered off and washed with water four times and 2 mL each time. The combined filtrate was titrated with 0.01 N NaOH using phenol red as indicator [27].

### 3.3. Preparation of acylals

Typical procedure for the synthesis of acylals (Scheme 2): The novel catalyst (25 mg) was added to a stirred solution of carbonyl compound (0.02 mol) and acetic anhydride (0.04 mol), then the reaction mixture was stirred at room temperature for the time as shown in Table 1 and Table 2. The reaction was monitored by TLC. After completion, the mixture was diluted with ethyl acetate and filtered. The organic layer was washed with 10% NaHCO_3_ solution and saturated solution of NaHSO_3_ and then dried over anhydrous Na_2_SO_4_. The solvent was evaporated under reduced pressure and the product purified by column chromatography on silica gel to give the corresponding products. All the compounds were characterized by GC–MS (Agilent 6890N GC/5973N MS, DM-5MS) and ^1^H-NMR(BRUKER, AVANCE 500 MHZ).

## 4. Conclusions

In conclusion, a novel heterogeneous strong acid catalyst has been found to be highly efficient for the formation of 1,1-diacetates from aliphatic and aromatic aldehydes. Operational simplicity without need of any solvent, exceptionally fast reactions, low cost of the catalyst used, high yields, excellent chemoselectivity, and applicability to large-scale reactions are the key features of this methodology.
